# Tailoring Pyro- and Orthophosphate Species to Enhance Stem Cell Adhesion to Phosphate Glasses

**DOI:** 10.3390/ijms22020837

**Published:** 2021-01-15

**Authors:** Nigel De Melo, Lauren Murrell, Md Towhidul Islam, Jeremy J. Titman, Laura Macri-Pellizzeri, Ifty Ahmed, Virginie Sottile

**Affiliations:** 1 School of Medicine, University of Nottingham, Nottingham NG7 2RD, UK; paxnd1@exmail.nottingham.ac.uk (N.D.M.); ezzlmp@exmail.nottingham.ac.uk (L.M.-P.); 2 Advanced Materials Research Group, Faculty of Engineering, University of Nottingham, Nottingham NG7 2RD, UK; emxlm1@exmail.nottingham.ac.uk (L.M.); ezami6@exmail.nottingham.ac.uk (M.T.I.); 3 Department of Applied Chemistry and Chemical Engineering, Faculty of Engineering, Noakhali Science and Technology University, Noakhali 3814, Bangladesh; 4 School of Chemistry, University of Nottingham, Nottingham NG7 2RD, UK; jeremy.titman@nottingham.ac.uk; 5 Department of Molecular Medicine, The University of Pavia, 27100 Pavia, Italy

**Keywords:** biomaterials, stem cells, phosphate-based glasses, cell culture, material degradation

## Abstract

Phosphate-based glasses (PBGs) offer significant therapeutic potential due to their bioactivity, controllable compositions, and degradation rates. Several PBGs have already demonstrated their ability to support direct cell growth and in vivo cytocompatibility for bone repair applications. This study investigated development of PBG formulations with pyro- and orthophosphate species within the glass system (40 − x)P_2_O_5_·(16 + x)CaO·20Na_2_O·24MgO (x = 0, 5, 10 mol%) and their effect on stem cell adhesion properties. Substitution of phosphate for calcium revealed a gradual transition within the glass structure from Q^2^ to Q^0^ phosphate species. Human mesenchymal stem cells were cultured directly onto discs made from three PBG compositions. Analysis of cells seeded onto the discs revealed that PBG with higher concentration of pyro- and orthophosphate content (61% Q^1^ and 39% Q^0^) supported a 4.3-fold increase in adhered cells compared to glasses with metaphosphate connectivity (49% Q^2^ and 51% Q^1^). This study highlights that tuning the composition of PBGs to possess pyro- and orthophosphate species only, enables the possibility to control cell adhesion performance. PBGs with superior cell adhesion profiles represent ideal candidates for biomedical applications, where cell recruitment and support for tissue ingrowth are of critical importance for orthopaedic interventions.

## 1. Introduction

In regenerative medicine, restoration of biological function can be aided by biomaterials created from metals, ceramics or polymers offering a variety of chemical structures, surface properties, degradation rates, and mechanical stiffness [1,2]. These properties allow for material functionalization to promote cell adhesion, support cell differentiation, exert anti-microbial effects, and assist drug delivery, among other applications [3,4,5]. Phosphate based glasses (PBGs) are biomaterials of particular interest due to their complete yet controllable resorption profiles which can be modified by simply altering the materials composition [6,7]. Glass is typically composed of network formers such as silicon or phosphate linked together through bridging oxygen atoms [8]. PBGs use phosphate (P_2_O_5_) as the network former, which can be structurally disrupted through the introduction of network modifier oxides which form non-bridging oxygen atoms [9]. The number of bridging oxygen atoms per tetrahedra, which define the Q^n^-speciation, are progressively broken down as network modifiers are added to the composition, decreasing connectivity within the glass [10]. Through modifications of the glass composition, it is entirely possible to influence a gradual decrease in structural connectivity (Q^3^ to Q^0^) which is denoted respectively as ultra-, meta-, pyro-, and orthophosphates [11,12,13].

When a higher percentage of P_2_O_5_ is incorporated in the composition, more than 50 mol%, the resulting glass possesses an ultraphosphate structure. However, when the P_2_O_5_ content remains at 50 mol% the resulting phosphate glass mainly contains Q^2^ (metaphosphate) species and when the P_2_O_5_ content is reduced further to below 50 mol% the glass transitions towards Q^1^ (pyrophosphate) species; where these formulations are referred to as invert glasses [9,13]. The glasses composed of higher amounts of phosphate content often degrade too fast for use as stable implants. Whereas invert glasses have the advantage of slower and more controlled degradation rates, with multicomponent glasses showing very little degradation after immersion in aqueous solutions [14]. Phosphate invert glasses consist of pyrophosphate (Q^1^) and orthophosphate (Q^0^) species, where upon eventual degradation it is anticipated that these two phosphate anions would be released. The key advantage of these ions are that they are found naturally within the body and can act towards beneficial support to regeneration and homeostasis of bone and other tissues [15].

It has been reported that when binding to hydroxyapatite (HA) which is composed of orthophosphate anions, inorganic pyrophosphate (PPi) can inhibit mineralisation by blocking further attachment and preventing the growth of crystals. While this could be problematic and disadvantageous for successful regeneration of bone architecture, pyrophosphate anions also contribute towards the homeostasis of bone [16,17]. The majority of extracellular PPi will interact with pyrophosphatases such as alkaline phosphatase (ALP) and tissue non-specific alkaline phosphatases (TNAP), enzymes which can be found bound to osteoblast cells and within matrix vesicles that are released [18]. Enzyme interactions cause PPi hydrolysis which consequently enables formation of orthophosphate (Q^0^) ions [19]. These orthophosphate ions are crucial for successful formation of bone HA crystals and are utilised in mineralisation of the collagen matrix previously laid down by the osteoblasts engaging in new bone formation [20]. This PPi degradation pathway is a promising avenue to explore for enhancing bone formation since it utilises naturally available mechanisms to generate two orthophosphate anions from each pyrophosphate molecule, thereby influencing the dynamic remodelling cycle.

As PBGs can incorporate various oxides as network modifiers, careful selection of specific oxides can confer cellular benefits when released, in addition to modifying glass properties [21]. For example, incorporation of calcium conveys the advantage of not only nucleating and mineralizing matrix through its release, but also directly influencing osteoblasts through calcium sensing receptors [22,23]. Magnesium has also been shown to play a key role in angiogenesis and formation of the ALP enzyme [24,25,26]. Phosphate species such as pyrophosphates have been demonstrated to promote ALP activity and gene expression [17]. Several studies have reported the supplementation of PBGs with strontium and zinc among other ions, demonstrating the variety of PBG compositions and applications [27,28,29]. Furthermore, the osteogenic effect of inorganic ions recently underlined the ability of PBG fibers to facilitate proliferation and osteogenic gene expression through the release of dissolution products [30]. Additional studies have demonstrated the biocompatibility and antimicrobial activity of PBG, and suggested their suitability for wound healing applications [31,32,33,34].

The controllable degradation rates of PBGs is a key asset to mimic the elevated levels of calcium and phosphate seen at sites of bone resorption, promoting regeneration of bone [15,35]. Additionally, PBGs can be manufactured to suit a variety of geometries. For example, PBGs in microsphere form allow for superior defect filling, scaffolding and flow properties, offering minimally invasive delivery routes [34,36,37]. A therapeutic product which is injectable, biocompatible, and can support osteogenic cells could prove highly effective to combat the degenerative nature of some bone diseases such as osteoporosis, potentially inhibiting or reversing bone loss subsequently resulting in reduced fracture risk [38]. To fulfil defect filling and tissue scaffolding roles, PBGs need to provide an ideal surface for cell adhesion. This study explores the tailoring of phosphate species in PBG formulations [34,36] to improve cell adhesion properties as it hypothesized that PBGs containing high pyro- and orthophosphate content would achieve superior results. As such, three PBGs of varying pyro- and orthophosphate content ratios were produced and used for human mesenchymal stem cell (MSC) culture to assess their ability to support cell adhesion in vitro.

## 2. Results

### 2.1. Glass Characterisation

To confirm the composition of the different PBGs produced, EDX analysis was performed on each formulation and the measurements for P40, P35 and P30 confirmed that the materials were within 1–4 mol% of the target formulation with a maximum deviation in percentage difference revealed to be 1.7 mol% (Table 1). P_2_O_5_ was substituted for the equivalent mol% CaO through the formulations, while the concentrations of MgO and NaO remained unchanged.

**Figure 1:**
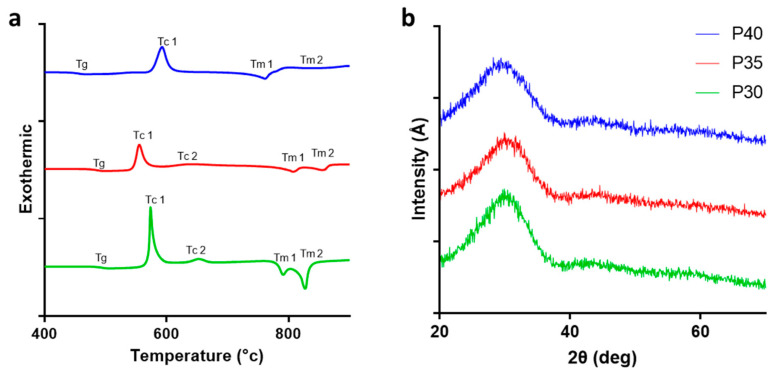
Glass characterization of P40, P35, and P30. (**a**) Thermal analysis traces via differential scanning calorimeter (DSC) of the phosphate-based glasses (PBG) formulations showing Tg, Tc, and Tm. (**b**) XRD spectra confirming the amorphous nature of P40, P35, and P30 glasses produced.

Thermal analysis was carried out via differential scanning calorimeter (DSC) analysis to determine the thermal properties of the glasses explored, in particular, the glass transition (T_g_), crystallisation (T_c_), and melting (T_m_) temperatures of each formulation as phosphate content was decreased. Figure 1a shows the DSC profiles of the three formulations and the T_g_, T_c_, and T_m_ values are presented in Table 2. Phosphate content substitution for calcium resulted in increases in T_g_ by 31 °C between P40 and P35 and a further increase of 9 °C between P35 and P30. Of the glasses tested, both P40 and P35 revealed a single crystalline peak with a T_c_ of 592 °C decreasing to 557 °C while P30 revealed a sharp single crystallisation peak at 578 °C and a smaller peak at 654 °C. These peaks indicated the possible presence of multiple crystalline phases existing within the respective glass formulations produced. The thermal processing window provides insight into the materials stability i.e., the resistance of a glass to crystallisation. Between P40 and P35 the processing windows were observed to have shortened dropping from 118 °C to 57 °C supporting the tendency towards crystallisation observed in low phosphate glasses as P30 also showed a processing window shorter than that of P40 at 68 °C.

X-ray powder diffraction (XRD) was carried out to confirm the nature of the formulations produced, which revealed the absence of crystalline peaks for all three formulations and the presence of a broad ‘halo’ peak characteristic of the amorphous nature of the glasses (Figure 1b).

**Figure 2:**
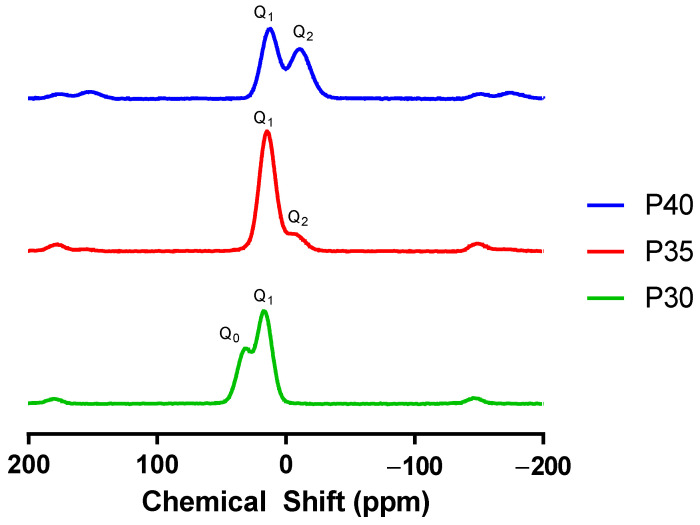
^31^P nuclear magnetic resonance (NMR) spectra of the P40, P35, and P30 formulations.

Nuclear magnetic resonance (NMR) analysis was used to confirm the distribution of different Q^n^ phosphate species within the three PBG formulations. As the phosphate content decreased from 40 mol% to 30 mol%, the phosphate species found within the glass structure decreased from Q^2^ and Q^1^, meta- and pyrophosphates, to Q^1^ and Q^0^, pyro- and orthophosphates. The NMR spectra for P40 showed two dominant peaks representing Q^1^ (−8.6 ppm) and Q^2^ (−22.8 ppm) units [9,39] (Figure 2).

P35 also demonstrated similar peaks identified as Q^1^ (7.4 ppm) and Q^2^ (−21.3 ppm) units. The slight shift in peaks associated with the Q^2^ species was suggested to be due to substitution of 5 mol% phosphate for calcium and a decrease in Q^2^ species content from 49% to 6% followed by equivalent increases in Q^1^ species from 51% to 94%. When the level of phosphate content was further decreased to 30 mol% in the P30 formulation, a shift from Q^2^ and Q^1^ to Q^0^ (3.3 ppm) fractions was observed, indicating presence of orthophosphate content, which was absent from the other two formulations explored revealing a Q^1^ to Q^0^ ratio of 61% and 39%, respectively (Table 3).

### 2.2. Cell Adhesion

**Figure 3:**
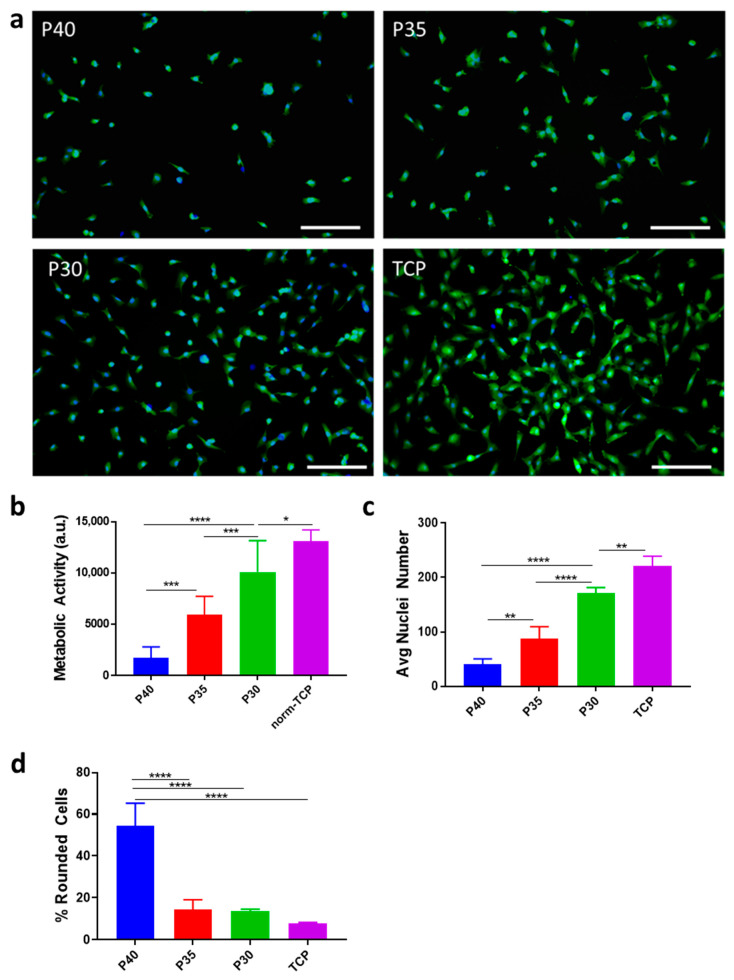
Cell adherence analysed 24 h after seeding on P40, P35, P30, and tissue culture plastic (TCP) surfaces. (**a**) Representative fluorescent images showing green fluorescence protein (GFP)-labelled mesenchymal stem cells (MSCs) (green) and Hoechst stained nuclei (blue). (**b**) Metabolic activity assay (norm-TCP shows activity for cells seeded onto TCP normalized to disc surface area). (**c**) Average nuclei count per field of view at 24 h. (**d**) Average percentage of rounded cells per field of view at 24 h. Scale bar = 250 μm. a.u.: arbitrary fluorescence units. * *p* < 0.05, ** *p* < 0.01, *** *p* < 0.001 and **** *p* < 0.0001. (*n* = 3).

An in vitro analysis was conducted to identify the impact of altering phosphate and calcium content of PBG on their capacity for stem cell adhesion and viability, comparing the effect of varying meta-, pyro-, and orthophosphate content. To this end, green fluorescence protein (GFP)-labelled MSCs were seeded onto discs (9 mm × 2 mm) made from the three PBG formulations, with tissue culture plastic (TCP) as a control, and cultured for 24 h. MSCs were able to attach to all three formulations tested, however, there were notable differences (Figure 3). 

As the phosphate content decreased from 40 mol% to 30 mol%, an increasing number of cells remained adhered to the glass disc surface (Figure 3a). The number of adherent cells increased in the order P30 > P35 > P40, and this was supported by results from the metabolic activity and nuclei count assays performed at 24 h, the latter showing a significant 4.3-fold increase in cells counted (Figure 3b,c). Furthermore, many of the cells on the P40 formulation displayed a rounded morphology, characteristic of cells with low substrate attachment [40]. By comparison, the proportion of these rounded cells decreased significantly on the P35 and P30 formulations (Figure 3d).

## 3. Discussion

Among the wide range of biomaterials being developed for orthopedic applications, PBGs offer significant advantages in terms of geometric and chemical versatility, which make them well-suited for regenerative medicine applications [27,34,41]. Importantly, PBGs can be produced as porous microspheres, which allows for injectable, minimally invasive delivery for defect filling applications while offering high surface area for enhanced cell adhesion and increased release kinetics [37,38]. While the use of various biocompatible coatings, such as collagen, can offer a means to ensure cell adhesion to biomaterials [42,43], the ability of specific PBG formulations to influence cell attachment without the use of ancillary biological components underlines their potential for clinical applications. As such, PBG microspheres are promising candidates for new orthopaedic therapies seeking to promote tissue repair [15], particularly in cases of bone loss where an increased risk of fracture is prevalent [44]. The high surface area and defect filling capacity of porous microspheres can promote cell-material interactions and tissue integration through and around microsphere clusters as previously observed in vitro [27] and in vivo [34]. This cell-material interaction naturally hinges on the capacity of the glass to promote cell adhesion and bone tissue formation.

To contribute towards the formation of new bone tissue during regeneration, phosphate anions as well as calcium and magnesium cations are essential. Within the natural cycle of bone resorption and formation, both pyro- and orthophosphates have an established role that can be directed towards the generation of new bone and HA crystallisation [45,46]. Inorganic pyrophosphates can act to modulate biomineralization, behaving both as an inhibitor for mineral formation and as a source of orthophosphates once hydrolysed by ALP [20,47]. In addition, they have been shown to stimulate the differentiation of pre-osteoblasts whilst also encouraging stimulation of ALP activity and extracellular matrix gene expression [17], and the presence of orthophosphate anions is directly linked to bone formation, as they are instrumental in the natural process for the mineralisation of collagen fibers which are laid down by osteoblasts during bone remodelling [19]. Phosphate-based glasses hold significant potential for bone regeneration, offering support for the biomineralisation process through their modifiable chemical composition and releasing beneficial ions at controllable rates in aqueous environments [48,49,50].

### 3.1. Material Characterisation

**Figure 4:**
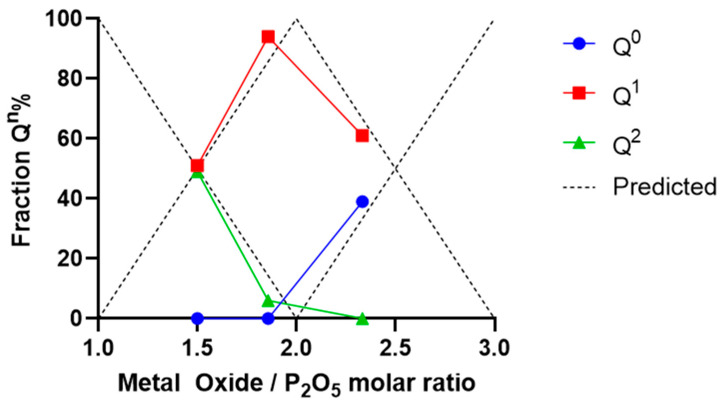
Alignment of P40, P35 and P30 to the predictions presented by Wazer and Fluck (1959) [57].

Compositional analysis of P40, P35, and P30 using EDX confirmed their composition to be within 1–4% of the target formulation. This is in line with other PBG investigated in the literature, particularly P40 [41]. No crystallisation peaks were observed for all three glass formulations, confirming their amorphous structure. Thermal analysis indicated a linear increase in T_g_ as phosphate content was substituted for calcium. This was likely due to the increase in divalent calcium cations, resulting in increased cross-linking of the phosphate chains within the glass structure [51,52,53]. This correlates with data presented by Franks et al. [54] who demonstrated a linear increase in T_g_ as calcium was added to the 45P_2_O_5_·(8 + x)CaO·(47 − x)Na_2_O (x = 2–32) PBG system, and by Ahmed et al. [55] who demonstrated a similar linear increase in T_g_ with calcium addition into the PBG system 50P_2_O_5_·(50 + x)CaO·(x)Na_2_O (x = 0–50). A similar shift in phosphate species was also reported in a study where the phosphate content of the quaternary glass CaO-P_2_O_5_-TiO_2_-Na_2_O was reduced from 55 mol% to 28 mol% [56]. The glasses that possessed a phosphate content between 55 mol% and 40 mol% demonstrated the phosphate species Q^2^ and Q^1^ while a further decrease in phosphate content to 35 mol% resulted in a glass that consisted of mainly Q^1^ pyrophosphates. Moreover, continued decreases in the phosphate content to 30 and 28 mol% resulted in glasses possessing only Q^1^ and Q^0^ phosphate units within the structure. The Q^n^ species distribution of all three PBGs aligned closely with the model predictions defined by Wazer and Fluck [57] as demonstrated by Figure 4.

### 3.2. Cell Adhesion

Cell adhesion was analysed using polished glass discs to evaluate the impact of the phosphate to calcium substitutions on the cell response to the material. All formulations were shown to support cell adhesion, with P30 demonstrating the highest capacity for cell adhesion and P40 demonstrating the lowest in comparison. These results are likely due to the combination of two factors, ion–cell interactions and material degradation.

Ion–cell interactions can occur through a number of mechanisms: (i) Calcium ions triggering the macromolecular calcium-sensing receptor (CaSR)/integrin complexes that promote adhesion [58] (ii) Direct divalent cation binding, particularly α-subunit containing integrins [59]. It is worth noting that in addition to calcium all the disc formulations used also contained magnesium, which is also a divalent cation, and could be capable of activating the low conformation state of the heterodimer integrins [60].

In addition to ion–cell interactions, material degradation properties are also likely to play a role in the results observed due to the impact degradation has on the local micro-environment, such as causing changes to pH and osmotic pressure. Abou Neel et al. [61] showed increased adhesion and proliferation rate of an osteosarcoma cell line (MG63) on PBG discs containing increasing mol% of titanium (0–5% in CaO·Na_2_O·P·Ti). On day 4, glasses without titanium content showed a significant decrease in proliferation compared to the formulation containing 5% titanium. Bitar et al. [62] also demonstrated increased adhesion and proliferation of human osteoblasts on PBG discs with increasing mol% of calcium through Na_2_O substitution for CaO (30–34% in CaO·Na_2_O·P_2_O_5_). Glasses with less than 40% CaO (in favour of increased Na_2_O) supported little to no cell adhesion after 24 h. A common correlation with these studies and the observations presented here, was an increase in cellular adhesion with decreasing glass solubility. The addition of titanium and substitution of Na_2_O for CaO would decrease the solubility of the respective glasses, as seen with the P_2_O_5_ for CaO substitution in this study. This data is further supported by a previous report [43] pointing to the deleterious nature of a rapidly degrading glass surface through scanning election microscopy and viability analysis of MSC, which demonstrated higher DNA content when the glass with the most CaO (40–48% CaO·2Na_2_O·50P) was used.

Studies investigating the degradation of invert glasses (37P_2_O_5_·29CaO ·10MgO·24Na_2_O) which took place over 56 weeks, revealed a cumulative weight loss of only 25% over one year in simulated body fluid [49]. Additionally, when compared to metaphosphate glasses, invert glasses demonstrated ion release rates that were more than an order of magnitude lower (2 mM vs. 80 mM phosphate) [63]. The P30 formulation tested in this study demonstrated increased pyro- and orthophosphate content when compared to P35 and P40 which consist of only pyro- and metaphosphates. As such, it is likely that P30 possesses favorable dissolution rates as observed in other invert glasses and therefore supported enhanced cell adhesion due to minimal changes to material surface integrity and pH/osmolarity. Navarro et al. [6] suggested that local variations in the liquid environment near the glass surface may be negatively impacting cells due to concentrated changes in pH and ionic levels. Skelton et al. [43] who showed increased cell adhesion in a formulation containing more calcium mol% (48 mol%) than other formulations in the 50P·(40 + x)CaO·(10 − x)Na_2_O glass system, suggested similar reasons for cell loss, citing potential contribution from factors such as surface integrity, ion–cell interactions and pH/osmolarity change at the material-cell interface. A study by Tošić et al. [64] using scanning electron micrograph images of an immersed glass surface, demonstrated disruption of the glass surface through the formation of pits followed by their expansion in size and number by 24 h. Naturally, a slower degrading glass would result in a more stable glass-medium interface. In addition to this, the breakdown of physical surface of the glass itself would likely impair adhesion events. The increased pyro- and orthophosphate content within the P30 formulation ultimately allowed for a more stable glass surface, promoting cell adhesion through the avoidance of the pH/osmolarity fluctuations suggested by other studies. One question of interest for future characterisation will be to what extent the cell attachment achieved on the PBG can in turn affect its rate of degradation.

### 3.3. Impact beyond Cell Adhesion

P30 demonstrated a significant increase in cell adhesion number in comparison to P35 and P40. While cell adhesion and therefore colony establishment on PBG are critical, particularly in patients with decreased stem cell counts, subsequent mechanisms to promote bone formation are of equal importance. Due to its composition, P30 demonstrated a high percentage of orthophosphates (~40%), the remaining structure made up of pyrophosphates that can be advantageous when used in bone regeneration, providing support for growth of HA crystals when hydrolysed by the osteoblast enzyme ALP, while stimulating osteoblast differentiation and metabolic activity [17,20]. In addition, the inorganic pyrophosphate structure possesses considerable similarity to the organic bisphosphonate drugs used in the treatment to prevent excessive bone resorption by osteoclasts. The backbone of the drugs are organic pyrophosphate molecules which are used as a “bone hook” to bind to existing HA whereupon the drugs are internalised by osteoclasts during resorption leading to cell death and an overall shift in bone homeostasis towards new bone formation [65,66]. Similarly, exposure to inorganic dicalcium pyrophosphates (1 × 10^−4^ M) has been shown to cause differential effects in bone cells specifically demonstrating an increase in osteoblasts, while the cell count for osteoclasts decreased; a response that was attributed to the cells having undergone apoptosis due to the incorporation of pyrophosphates [66]. To determine the benefits of pyrophosphate inclusion, studies have concluded that its addition to calcium phosphate cement implants allowed the pyrophosphate to act as a substrate for osteoblast membrane ALP and encouraged the formation of HA over 12 months [20]. Moreover, different concentrations of pyrophosphate have been shown to stimulate pre-osteoblast differentiation and increase the peak ALP activity. At the highest concentrations (100 µM), increased expression of osteogenic genes including Collagen 1, ALP, and Osteocalcin was observed indicating the influence of pyrophosphate upon osteoblast differentiation [17,20]. It will thus be of interest to monitor osteogenic markers, including ALP, in cells exposed to P30.

Additionally, calcium orthophosphates have been incorporated as part of numerous biomaterials due to their similarity to naturally occurring HA in bone tissue [34]. Inclusion of calcium orthophosphates in new biomaterials for bone applications represents a nontoxic component that would exhibit bioactive behaviour and allow for osteointegration, aiding integration with living tissue [67]. When combined with collagen, calcium orthophosphates represent an effective osteoconductive material enhancing osteoblast differentiation and accelerating osteogenesis [68].

The modulation of their composition naturally changes the structure of phosphate-based glass and can influence the phosphate species present within the material that are eventually released as anions and made available to the local environment. The changes in cell response reported here highlight how the adhesion profile of a glass can be controlled and improved through tailoring of the ionic species present within its composition. The present in vitro results call for complementary investigations of in vivo parameters representing the complex nature of the bone environment which consists of numerous cell types, metabolites, pH and ionic conditions that can further influence cell adhesion, and will inform future clinical trials. Based on P30′s strong cell adhesion profile, future studies will evaluate whether this new material may also enhance osteoblast differentiation and support bone formation in vivo.

## 4. Materials and Methods

The reagents used in this study were purchased from ThermoFisher Scientific (Loughborough, UK) unless otherwise stated.

### 4.1. PBG Production

The precursors P_2_O_5_, NaH_2_PO_4_, CaHPO_4_, and MgHPO_4_·3H_2_O (Sigma Aldrich, Gillingham, UK were used to fabricate PBGs of the system (40 − x)P_2_O_5_·(16 + x)CaO·20Na_2_O·24MgO, where x is 0, 5 and 10 mol% (Table 4) [38]. The precursors were mixed and placed within a 100 mL Pt/5% Au crucible (Birmingham Metal Company, Birmingham, UK). The crucible was then placed in a 350 °C furnace for 30 min, ramped to 460 °C for 30 min (10 °C/min) then held for 30 min. A second ramp (10 °C/min) to 1150 °C was then reached and held for 90 min. The melts were poured onto a steel plate. A splat quench technique was performed on P30 to avoid surface crystallisation effects.

To manufacture microspheres, a planetary zirconia ball mill (Retsch Planetary Mill PM100) was used to grind the quenched glass for 3 min at 350 rpm at least 3 times. Stainless steel sieves (VWR International, Lutterworth, UK) were then used to sieve the glass particles into a size range of 63–125 μm. Porous PBG microspheres were prepared by mixing the glass powders with CaCO_3_ in a 1:3 ratio before the flame spheroidisation process using a thermal spray gun. After cooling, the microspheres were collected from the cooling trays and porous microspheres were washed in 5 mM acetic acid for 2 min followed by a deionized water wash for 5 min and then dried in a 50 °C oven for 24 h.

To produce glass discs, molten glass from each formulation was poured into graphite moulds with 9 mm diameter rod cut outs and held at 10 °C above the glass transition temperature (T_g_) for each composition for 1 h before being allowed to cool to room temperature overnight. After cooling, the glass rods were removed from the moulds and cut using a diamond saw blade (Low Speed Diamond Wheel Saw Model 650 CE, South Bay Technology Inc., San Clemente, CA, USA) to 2 mm width. Metallographic abrasive paper (P240, P400, P800 and P1200), followed by 6 µm and 1 µm polishing pads and diamond pastes were used to polish the surfaces of the glass discs. Industrial methylated sprit was used as a lubricant. Once prepared, the discs were sterilized through two 15-min washes in ethanol 70% and allowed to dry for 24 h at room temperature in a sterile environment.

### 4.2. Energy-Dispersive X-ray Spectroscopy

Microspheres were embedded in resin (EpoFix Resin and Epofix Hardener, Struers, Cleveland, OH, USA) before 10 nm carbon coating. Samples were then analysed at a 10 mm working distance using a 10 kV beam using a XL30 SEM (FEI/Philips, Amsterdam, The Netherlands).

### 4.3. Thermal Analysis

A Differential Scanning Calorimeter (DSC, TA instruments, New Castle, DE, USA, Q600) was used to determine the glass transition (T_g_), onset crystallisation (Tc_ons_), peak crystallisation (T_c_), and glass melting temperature (Tm) points of the produced glass formulations. The processing window for the different glasses was determined using the following equation:

Processing window = (Tc_ons_ − T_g_)

20 mg of powdered glass samples were added to a platinum sample pan and heated from room temperature to 1100 °C at a ramp of 10 °C/min. A second blank platinum pan was used for a baseline to correct the thermal traces of the samples.

### 4.4. X-ray Powder Diffraction

XRD was used to confirm the amorphous nature of the PBGs produced. Ground glass powder samples were run using a DVA-Da Vinci Advantage over a 2 theta range from 5 degrees to 70 degrees with a 0.02 degree step size and a step time over 4 s.

### 4.5. Nuclear Magnetic Resonance

NMR was used to resolve the distribution of different Q^n^ species within the PBGs produced. Solid-state ^31^P NMR spectra were recorded for the PBGs at room temperature using a Varian Chemagnetics Infinity plus spectrometer operating at a Larmor frequency of 121.47 MHz using a 4 mm magic angle spinning (MAS) probe spinning at 12 kHz. The ^31^P π/2 pulse duration was 3.7 µs, the spectral width was 100 kHz and the acquisition time was 10.24 ms. Chemical shifts are quoted relative to 85% H_3_PO_4_. Prior to acquiring ^31^P spectra the spin-lattice relaxation time T_1_ was determined for each sample by saturation recovery. Saturation was achieved by 100 ^31^P π/2 pulses spaced by delays of 5 ms with recovery delays of up to 1000 s. Quantitative ^31^P NMR spectra required relaxation delays (5 T_1_) of between 500 s and 650 s. The resulting spectra were deconvoluted into a set of Gaussian line shapes which were integrated in order to quantify the proportions of the different Q environments in the sample. First-order MAS sidebands were included in the analysis.

### 4.6. Cell Culture

For the direct culture study, a human immortalized bone marrow-derived mesenchymal stem cell line (MSC) labelled with Green Fluorescence Protein (as previously described in Harrison et al., 2017; Macri-Pellizzeri et al., 2018) was seeded onto sterilized discs at a density of 8000 cells/cm^2^ in standard cell culture medium (SM) (low glucose DMEM supplemented with 10% Foetal calf serum (FCS), 1% penicillin and streptomycin, 1% L-Glutamine, and 1% non-essential amino acids) and cultured at 37 °C and 5% CO_2_ for 24 h. Cells seeded on tissue culture plates (TCP) were used as a control.

### 4.7. Metabolic Activity Assay

After 24 h of culture, the cell-seeded discs of each formulation were transferred to a new 48-well plate and the metabolic activity of seeded cells was analyzed using the Presto blue Cell Viability Reagent following the manufacturer’s instructions. Briefly, a working solution of 10% of Presto Blue reagent in SM was prepared and 300 μL was added to the cells after one PBS wash. After 45 min incubation in the dark at 37 °C and 5% CO_2_, 250 μL of the solution were then transferred to a new 96-well plate for the measurement of fluorescence at 560 nm and 590 nm the excitation and emission wavelengths, respectively using a plate reader Infinite 200 (Tecan, Reading, UK). Values were normalized to the surface area to allow comparison with cells cultured on TCP.

### 4.8. Nuclear Staining and Imaging

After 24 h in culture, live cell nuclei were stained using Hoechst 33258 (Sigma-Aldrich, Gillingham, UK) at 20 μg/mL in SM for 1 h at 37 °C. Bright field images were taken using a Nikon Eclipse TS100 microscope, and fluorescent images were taken on a Nikon Eclipse TS2 microscope. Cell nuclei and cells displaying a rounded morphology were manually counted from four fields of view per disc using NHI ImageJ 1.8.0. (https://imagej.nih.gov/).

### 4.9. Statistical Analysis

Results for the metabolic activity assay and nuclei counts are presented as mean ± standard error of the mean (SEM). One-way analysis of variance with Turkey’s multiple comparison test was used. A 95% confidence level was considered significant. Graphs and statistical analysis were performed using GraphPad PRISM 7.04 software package (https://www.graphpad.com/, GraphPad Software, San Diego, CA, USA). * *p* < 0.05, ** *p* < 0.01, *** *p* < 0.001 and **** *p* < 0.0001.

## 5. Perspectives

The PBG discs demonstrated a linear response of increasing stem cell adhesion as phosphate was substituted for calcium, with P30 demonstrating a significant increase in adherent cells compared to the P40 formulation. This trend in cell adhesion was linked to the high concentration of pyro- and orthophosphate content in P30, resulting in a more stable and favorable glass surface for cell adhesion when compared to both P35 and P40. The initial phase of progenitor cell seeding and culture establishment in in vivo applications is of critical to initiate the tissue repair process. This study demonstrates the successful tailoring of PBG formulation through increases in pyro- and orthophosphate species within the glass to enhance stem cell adhesion and support cell culture. In addition to providing a supporting surface for cell adhesion, the potential benefits of inorganic ions and phosphate species released from the PBG represent a promising resource for orthopaedic and tissue repair applications.

## Figures and Tables

**Table 1:** EDX measurements of glass compositions against target formulation (mol%) ± standard error of the mean (SEM).

		P_2_O_5_ (mol%)	CaO (mol%)	MgO (mol%)	NaO (mol%)
P40	Target	40	16	24	20
	Measured	39.4 ± 0.7	16.6 ± 0.6	24.0 ± 0.6	20.0 ± 0.7
P35	Target	35	21	24	20
	Measured	34.0 ± 0.4	25.5 ± 1.7	21.9 ± 1.0	18.7 ± 0.7
P30	Target	30	26	24	20
	Measured	28.2 ± 0.3	24.3 ± 0.7	26.0 ± 0.5	21.6 ± 0.5

**Table 2:** Glass transition (T_g_), onset of crystallisation (T_c_), melting temperature (T_m_), and calculated thermal processing window (Tc_onset_) for P40, P35, and P30.

	T_g_ Onset	T_c_ Onset	T_c_ 1	T_c_ 2	T_m_ 1	T_m_ 2	Window Tc_onset_ − T_g_
P40	445	563	592		761	838	118
P35	476	533	557	637	805	853	57
P30	485	553	578	654	790	825	68

**Table 3:** Distribution of Q^n^ species across PBG formulations.

	Q^0^	Q^1^	Q^2^
P40	0	51	49
P35	0	94	6
P30	39	61	0

**Table 4:** Glass composition (mol%).

Glass Code	P (mol%)	Ca (mol%)	Mg (mol%)	Na (mol%)
P40	40	16	24	20
P35	35	21	24	20
P30	30	26	24	20

## Data Availability

Data is contained within the article.

## Abbreviations

ALP	alkaline phosphatase
CaSR	Calcium-sensing receptor
DSC	Differential scanning calorimeter
FCS	Foetal calf serum
GFP	Green fluorescence protein
HA	Hydroxyapatite
MSC	Mesenchymal stem cell
NMR	Nuclear magnetic resonance
PBG	Phosphate based glass
PPi	Inorganic pyrophosphate
SEM	Standard error of the mean
T_c_	Glass crystallisation temperature
TCP	Tissue culture plastic
T_g_	Glass transition temperature
T_m_	Glass melting temperature
TNAP	tissue non-specific alkaline phosphatases
XRD	X-ray powder diffraction
